# What is a star worth to Medicare beneficiaries? A discrete choice experiment of hospital quality ratings

**DOI:** 10.1093/haschl/qxad085

**Published:** 2023-12-12

**Authors:** Logan Trenaman, Mark Harrison, Jeffrey S Hoch

**Affiliations:** Department of Health Systems and Population Health, School of Public Health, University of Washington, Seattle, WA 98195, United States; Collaboration for Outcomes Research and Evaluation, Faculty of Pharmaceutical Sciences, University of British Columbia, Vancouver, BC V6T 1Z3, Canada; Centre for Advancing Health Outcomes, Providence Health Care Research Institute, Vancouver, BC V6Z 1Y6 Canada; Center for Healthcare Policy and Research, University of California, Davis, Sacramento, CA 95817, United States; Division of Health Policy and Management, Department of Public Health Sciences, University of California, Davis, Davis, CA 95616, United States

**Keywords:** willingness to pay, patient preferences, quality of care, quality reporting, hospital report cards

## Abstract

Hospital quality ratings are widely available to help Medicare beneficiaries make an informed choice about where to receive care. However, how beneficiaries’ trade-off between different quality domains (clinical outcomes, patient experience, safety, efficiency) and other considerations (out-of-pocket cost, travel distance) is not well understood. We sought to study how beneficiaries make trade-offs when choosing a hypothetical hospital. We administered an online survey that included a discrete choice experiment to a nationally representative sample of 1025 Medicare beneficiaries. On average, beneficiaries were willing to pay $1698 more for a hospital with a 1-star higher rating on clinical outcomes. This was over twice the value of the patient experience ($691) and safety ($615) domains and nearly 8 times the value of the efficiency domain ($218). We also found that the value of a 1-star improvement depends not only on the quality domain but also the baseline level of performance of the hospital. Generally, it is more valuable for low-performing hospitals to achieve average performance than for average hospitals to achieve excellence.

## Introduction

In 2005, the Centers for Medicare and Medicaid Services (CMS) began public reporting of hospital quality measures through the Hospital Compare platform.^1^ The goal was to enhance transparency and support consumer choice as a means to improve the quality, efficiency, and accountability of the health care system. Subsequent policy reforms, including the 2010 Patient Protection and Affordable Care Act, have strengthened quality measurement and reporting.^2^

There is evidence that patients are more likely to choose hospitals with higher-quality ratings, and a considerable portion of this literature has focused on Medicare.^3-7^ For example, Pope^3^ found that the average Medicare hospital experienced a 5% change in non-emergency patient volume based on a change in quality rankings from the US News and Hospital Report. Chandra et al^6^ found evidence that higher-quality Medicare hospitals are rewarded with a greater market share, and that patient demand is an important contributor to this effect.

The CMS's Care Compare website provides a wide range of information to help patients compare hospitals in their area.^8^ This includes an overall star rating, which summarizes each hospital's performance across different domains of quality, and a patient-experience star rating, which is a summary of the hospital's performance on the domains of the Hospital Consumer Assessment of Healthcare Providers and Systems (HCAHPS) survey. The website also allows users to compare hospitals on more granular data, including quality indicators related to timely and effective care, complications and deaths, unplanned readmissions, maternal health, psychiatric services, and payment and the value of care provided.^8^

While public reporting of quality measures is commonplace, the preferences of Medicare beneficiaries for different aspects of hospital quality are not well understood.^9^ The purpose of this study is to describe how Medicare beneficiaries trade off between aspects of hospital quality, travel distance, and out-of-pocket cost, and to use this information to estimate how much individuals would be willing to pay or how far they would be willing to travel for higher rated hospitals.

## Data and methods

This analysis is part of a study that used an online survey that included a discrete choice experiment (DCE) to understand which aspects of hospital quality are most important to Medicare beneficiaries. We previously reported on the relative importance of different domains of hospital quality from the perspective of Medicare beneficiaries (eg, clinical outcomes vs patient experience) and the impact of using beneficiary preferences on value-based payment incentives.^10^ Importantly, these data also provide insight into how Medicare beneficiaries trade off between different levels of quality (eg, 3-star vs 4-star ratings), cost, and distance, and can be used to estimate how much beneficiaries are willing to pay or how far they are willing to travel for higher rated hospitals.

### Discrete choice experiment methodology

A DCE is a survey-based approach to elicit preferences.^11^ In a DCE patients are presented with hypothetical scenarios (called “choice sets”) consisting of 2 or more alternatives, which are described using several “attributes” (eg, cost), and corresponding “levels” (eg, $10, $50, $100). In each choice set, respondents are asked to choose their preferred alternative. This approach assumes that respondents choose the alternative that maximizes their utility (a concept which represents value), and that the utility of an alternative depends on the utility associated with its attributes and levels.^12^ This enables the estimation of the relative importance of attributes and levels and describes the extent to which people are willing to make trade-offs.

### Study design and sample size

**Figure 1.**
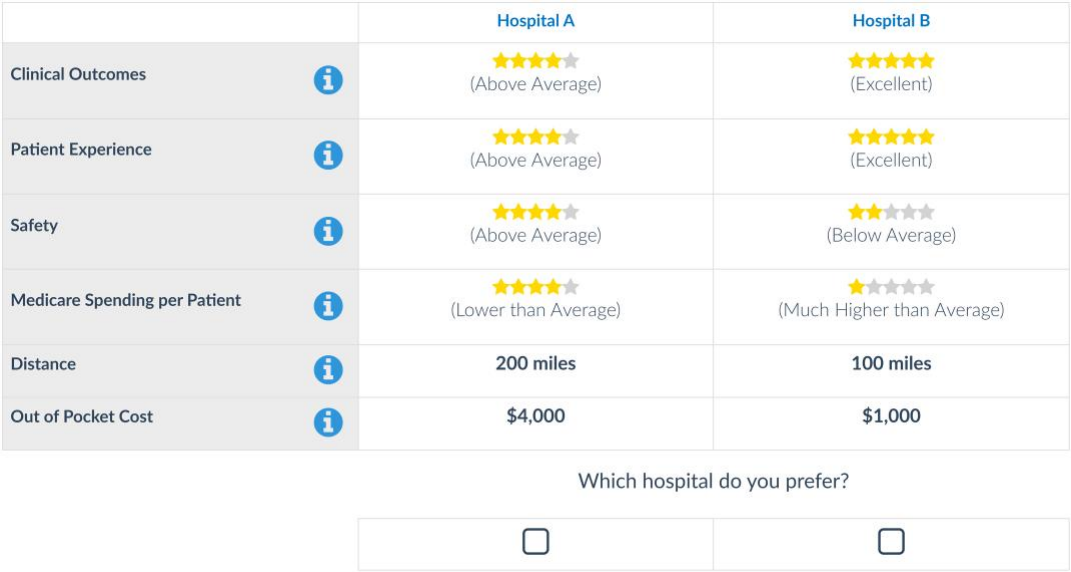
An example discrete choice experiment choice task.

We conducted a DCE through an online survey that asked a nationally representative sample of Medicare beneficiaries to choose between 2 hypothetical hospitals (Figure 1). Hospitals were described using 6 attributes. Four attributes described different aspects of quality and were derived from the quality domains of Medicare's Hospital Value-Based Purchasing Program: clinical outcomes, patient experience, safety, and efficiency (termed “Medicare spending per patient”). Each of the 4 quality attributes were described using 5 levels corresponding to Medicare's 5-star rating system. Descriptive labels for the star ratings were taken from Medicare's patient-facing website and ranged from “poor” to “excellent” for clinical outcomes, patient experience, and safety, and from “much higher than average” to “much lower than average” for efficiency.^10^ The use of stars was supported by our qualitative research, which included focus groups and think-aloud interviews. Aside from quality, patients value convenience and cost—and may prefer a hospital that is closer to their home or one with a lower out-of-pocket payment. Therefore, 2 attributes representing the distance from home to the hospital (10, 100, or 200 miles) and out-of-pocket cost for the hospital stay ($200, $1000, or $4000) were included. Levels for the distance and out-of-pocket cost attributes were derived from a previous DCE, which used star ratings.^9^ The out-of-pocket cost levels were based on the mean Medicare Part A inpatient deductible, while distance levels were meant to reflect different methods of travel. Our qualitative research suggested that these levels were reasonable to Medicare beneficiaries, and our pilot data confirmed that respondents were trading across levels.

With four 5-level and two 3-level attributes, there were over 5000 potential hospital profiles (54 × 32 = 5625). Presenting all possible combinations of hospital profiles (a “full factorial” design) was unfeasible, so we generated a design that included a subset (a “fractional factorial” design). We selected choice sets using the *dcreate* Stata algorithm (StataCorp, College Station, TX), which accounts for the fact that some choice sets are more informative than others (D-efficiency criterion).^13^ Our final design included 45 questions that were “blocked” into 5 versions, each with 9 questions (Table S1). The DCE was embedded in an online survey, which was refined through focus groups and think-aloud interviews with Medicare beneficiaries and pilot testing in a sample recruited through Amazon's Mechanical Turk.^14^

Participants were asked to provide online informed consent, introduced to the choice task, and provided with information on the 6 attributes and levels. There were 2 warm-up questions that were not scored, followed by 1 randomly assigned block of 9 DCE questions. For each question, participants were asked “Which hospital do you prefer?”

### Subject enrollment and data collection

Medicare beneficiaries were recruited through Ipsos KnowledgePanel, the largest online panel that is representative of the US population.^15^ KnowledgePanel uses an address-based sampling methodology that is based on the US Postal Service database, meaning that it covers all US households, even those without a phone. Panelists must be recruited to join and are provided with a computer and internet access if needed. KnowledgePanel has been widely used in health research,^16,17^ including for DCEs.^18,19^ Our target population included pre-identified panellists who were covered by Medicare and subsequently re-confirmed their coverage. We used stratified random sampling to identify a representative sample of adult Medicare beneficiaries according to age, sex, and race/ethnicity based on Medicare enrollment statistics for 2019, with oversampling in 3 racial groups (Black or African-American; Asian/Pacific Islander; Hispanic). The survey was fielded between March 15 and 29, 2022. A total of 1835 individuals were invited to participate, with email reminders sent to nonresponders on day 3.

### Data analysis

Our primary analysis used a mixed logit model. The advantage of the mixed logit model is that it accounts for preference heterogeneity by allowing the parameters to vary from one individual to another. We fitted 2 main effects models. Model 1 treated all attributes as continuous. Model 2 treated all quality attributes as categorical with the 1-star (lowest) level serving as the reference level. To ensure that our responses were representative of the Medicare population, we weighted respondents to match the characteristics of the Medicare population using sample weights generated using “raking” (also known as “iterative proportional fitting”).^20^ We sought to match the following distributions of the age 18-and-over US population with annual Medicare health insurance coverage: race-ethnicity by age group, gender by age group, census region, metropolitan status, and household income. The benchmarks were obtained from the 2021 March Current Population Survey Annual Social and Economic Supplement.^10^

We report trade-offs between the quality attributes and money (ie, willingness to pay [WTP]) and travel distance (ie, willingness to travel [WTT]). We estimated WTP and WTT using the mixed logit model in the WTP space (Stata: *mixlogitwtp*).^21^ The coefficient for all attributes were assumed to be random. Non-monetary and monetary attributes were assumed to be normally and log-normally distributed, respectively. For model 1, WTP/WTT is interpreted as how much respondents would be willing to pay or how far respondents would be willing to travel, on average, for a hospital with a 1-star higher quality rating. For model 2, WTP/WTT indicates how much respondents would be willing to pay or how far they would be willing to travel, on average, for a hospital with that star rating, relative to a 1-star hospital.

## Results

A total of 1025 Medicare beneficiaries completed the survey. Participants’ characteristics are summarized in Table 1. Table S2 outlines the key demographic characteristics of our unweighted and weighted samples and the Medicare population. Detailed results describing WTP and WTT from the mixed logit model for model 1 are summarized in Tables S3 and S4, respectively. All main effects of the attributes were statistically significant and in the expected direction. For example, model 1 found that the coefficients for quality attributes were positive, indicating that respondents preferred hospitals with higher quality ratings, while distance and out-of-pocket cost attributes were negative, indicating that respondents preferred hospitals that were closer to their home and cost less out-of-pocket.

**Table 1.** Respondent characteristics.

	*n*	%
Age group		
18–34 y	8	1
35–44 y	22	2
45–54 y	37	4
55–64 y	79	8
65–74 y	565	55
75+ y	314	31
Gender		
Female	518	51
Male	507	49
Race		
White	717	70
Black or African-American	211	21
Asian	63	6
American Indian or Alaska Native	11	1
2+ Races	23	2
Ethnicity		
White, non-Hispanic	535	52
Black, non-Hispanic	206	20
Other, non-Hispanic	66	6
2+ Races, non-Hispanic	14	1
Hispanic	204	20
Highest educational attainment		
No high school diploma or GED	71	7
High school graduate (high school diploma or the equivalent GED)	274	27
Some college or Associate’s degree	328	32
Bachelor's degree	163	16
Master's degree or higher	189	18
Household income		
Less than $10 000	23	2
$10 000 to $24 999	145	14
$25 000 to $49 999	246	24
$50 000 to $74 999	194	19
$75 000 to $99 999	135	13
$100 000 to $149 999	147	14
$150 000 or more	135	13
Marital status		
Divorced	143	14
Never married	118	12
Now married	618	60
Separated	19	2
Widowed	127	12
Metropolitan area status		
Metropolitan	901	88
Nonmetropolitan	124	12
Region		
Midwest	188	18
Northeast	160	16
South	439	43
West	238	23
Employment status		
Not working	869	85
Working full-time	49	5
Working part-time	107	10
Health insurance status		
Medicare, for people 65 and older, or people with certain disabilities	1025	100
Medicaid, Medical Assistance, or another government-assistance plan	93	9
Insurance through a current or former employer or union	179	17
Insurance purchased directed from an insurance company	141	14
TRICARE or other military health care	50	5
VA (enrolled for VA health care)	47	5
Indian Health Service or any other type of health insurance or health coverage plan	48	5

*n* = 1025. Abbreviations: GED, General Educational Development; VA, Veterans Affairs.

We found that, on average, Medicare beneficiaries would be willing to pay an additional $1698 (95% CI: $1483 to $1912) or travel 185 miles further (95% CI: 155 to 215 miles) for a hospital with a 1-star higher rating on clinical outcomes (Tables S3 and S4). Patient experience was the second most valuable quality domain (WTP: $691; 95% CI: $587 to $794; WTT: 77 miles; 95% CI: 63 to 91 miles) followed by safety (WTP: $615; 95% CI: $497 to $733; WTT: 62; 95% CI: 49 to 76) and efficiency (WTP: $218; 95% CI: $133 to $302; WTT: 20 miles; 95% CI: 12 to 28 miles). Our results also indicated that, on average, respondents were willing to pay approximately $825 (95% CI: $652 to $997) to avoid travelling 100 miles further. Conversely, respondents were willing to travel approximately 123 miles further (95% CI: 98 to 149 miles) to avoid paying $1000 out-of-pocket.

Respondents' WTP/WTT for model 2, which treated quality attributes as categorical, are reported in Table 2 and shown in Figure S1. Full-model results for WTP and WTT are presented in Tables S5 and S6, respectively. For clinical outcomes and patient experience the mean WTP/WTT for 1-star improvements to 2, 3, and 4 stars (from 1, 2, and 3 stars, respectively) were comparable, while the improvement to 5 stars (from 4 stars) was considerably less valuable. For example, respondents were willing to pay about $1628 (95% CI: $1164 to $2072) for a hospital with a 2-star (compared to 1-star) rating on clinical outcomes. In contrast, a 1-star improvement from a 4- to 5-star rating on clinical outcomes was only valued at approximately $300, as respondents' mean WTP for hospitals with 4- and 5-star ratings on clinical outcomes were $5419 (95% CI: $4750 to $6088) and $5720 (95% CI: 4984 to $6457), respectively (Table 2). For efficiency, our results suggest that respondents took a dichotomous view on hospital performance, differentiating between those that were either below average and average or above.

**Table 2.** Medicare beneficiaries' willingness to pay (WTP) and willingness to travel (WTT) for hospital quality ratings, model 2 (categorical).

	WTP, $			WTT, miles		
	β	2.5%	97.5%	β	2.5%	97.5%
Clinical outcomes						
One-star	Reference			Reference		
Two-star	$1618	$1164	$2072	187	129	244
Three-star	$3654	$3120	$4187	431	343	519
Four-star	$5419	$4750	$6088	636	507	765
Five-star	$5720	$4984	$6457	695	556	833
Patient experience						
One-star	Reference			Reference		
Two-star	$563	$188	$938	70	25	115
Three-star	$1312	$844	$1780	164	107	220
Four-star	$2041	$1528	$2553	217	154	281
Five-star	$2361	$1934	$2788	280	210	351
Safety						
One-star	Reference			Reference		
Two-star	$1131	$751	$1510	149	91	207
Three-star	$1898	$1481	$2314	248	182	313
Four-star	$1833	$1395	$2271	225	154	297
Five-star	$2655	$2157	$3153	274	193	355
Efficiency						
One-star	Reference			Reference		
Two-star	$23	−$319	$365	5	−35	44
Three-star	$600	$254	$947	107	63	151
Four-star	$561	$178	$945	57	15	99
Five-star	$747	$374	$1119	81	34	128
Distance (100 miles)	−$849	−$1038	−$660			
Cost ($1000)				−123	−149	−98
	Obs: 18 450 LL: −4387.57 AIC: 8847.14 BIC: 9128.76			Obs: 18 450 LL: −4303.72 AIC: 8679.44 BIC: 8961.06		

Abbreviations: AIC, akaike information criterion; BIC, bayesian information criterion; LL, log-likelihood; Obs, observations.

## Discussion

We examined Medicare beneficiaries' trade-offs between quality ratings, cost, and travel distance, providing estimates of how much beneficiaries are willing to pay and willing to travel to access higher rated hospitals. Our results indicate that there is variation in the value of different domains of hospital quality, and that the value of a 1-star improvement in quality depends on the quality domain and the baseline performance of the hospital.

Clinical outcomes were the most valuable quality domain, with respondents willing to pay an average of nearly $1700 for a hospital with a 1-star higher rating. A previous DCE focused on quality-metrics hospital choice for hip and knee replacement in Baltimore found that attributes related to clinical outcomes and safety (postoperative pain, complication rates, infections) were more important than those related to patient experience (cleanliness, overall rating, understandable discharge instructions).^22^ We found that Medicare beneficiaries were willing to pay an additional $218 for a hospital with a 1-star higher rating on efficiency. There are several potential explanations. Beneficiaries may value a more efficient hospital because it means the hospital can provide more care to more patients, or because it supports the financial sustainability of Medicare. Efficiency may also serve as a signal for other aspects of quality that are not captured by the other quality domains. For example, it may signal a hospital's commitment to limit unnecessary tests, treatments, and procedures.

Prior research has used the DCE methodology to explore trade-offs between quality ratings, cost, and travel distance when choosing between health care providers. However, comparisons are challenging given differences in the decision context, study population, the number of quality attributes, their scope, and the range and description of their respective levels. That said, Koch-Weser et al^23^ estimated that WTP for a 4-star (compared with 3-star) medical center was $621 for maternity care, $1983 for knee-replacement surgery, and $2735 for colon cancer. The authors noted that the variation in WTP across the different conditions likely reflects an increasing emphasis on quality when seeking care for conditions that are perceived to be more severe. Schwartz et al^9^ conducted a DCE to understanding trade-offs between hospital and physician quality ratings and cost and distance for patients considering total joint arthroplasty. On average, patients were willing to pay an additional $2600 for a hospital with a 1-star higher rating, and an additional $3150 for a physician with a 1-star higher rating. Further, Schwartz et al^9^ estimated that respondents were willing to pay approximately $11.50 to avoid travelling an additional mile, which was close to our estimate of $8.50. The slightly higher WTP reported by Schwartz et al may be explained by the study population, as 45% of participants reported an annual household income of $100 000 or more, compared with 27% in our sample.

We found evidence that the effects of quality on hospital choice are not linear. For example, for the clinical outcomes and patient experience domains, Medicare beneficiaries were willing to pay more or travel further to move from 1- to 2-star hospitals than from a 4- to 5-star hospital. The marginal WTP to move from a 4- to 5-star hospital on clinical outcomes was approximately $300, compared with an average of $1800 for the other three 1-star improvements. For the patient experience domain, the marginal WTP/WTT for a 4- to 5-star rating was less than half of the average value of the other three 1-star improvements. This finding of an asymmetric response to quality ratings is consistent with previous research.^24^ In exploring the impact of quality report cards for cardiac bypass surgery in New York, Cutler et al^4^ found that low-quality scores were associated with a significant decrease in demand, but there was no comparable increase in demand associated with a high-quality score. In the same context, Dranove and Sfekas^25^ found that low-quality scores made patients less likely to choose a hospital, while high-quality scores did not have an impact. The finding of an asymmetric response to quality ratings has implications for quality-improvement activities and can help hospitals target quality-improvement initiatives to aspects of care that matter most to Medicare beneficiaries. For example, our results suggest that, for clinical outcomes and patient experience, it is more valuable for hospitals that are performing poorly to achieve average performance than it is for above-average hospitals to achieve excellence. Other quality domains exhibited a “plateau” in the WTP/WTT, suggesting that there may be a threshold for what constitutes “acceptable” quality. This was most pronounced for the efficiency domain, where beneficiaries valued hospitals that achieved average performance (3 stars) similarly to those with above-average performance (4 and 5 stars).

We previously described how understanding the relative importance of quality domains from beneficiaries (ie, in a representative sample) could be used to align the incentives within value-based payment programs with quality as defined by patients.^10^ However, understanding the relative importance of quality domains also has implications for supporting patient choice. The 5-star ratings on the CMS's Care Compare website are often based on equally weighting the composite measures; however, different beneficiaries have different perspectives on quality. Barclay et al^26^ found that changing 2021 Hospital Compare domain weights for the overall star ratings would result in one-quarter of hospitals receiving a different star rating. Additional evidence has shown that changing domain weights can change the relative ranking of providers within a given market.^27^ For example, Mukamel et al^28^ allowed Medicare beneficiaries to construct their own composite 5-star ratings for nursing homes, including selecting the quality measure and specifying the associated weights, which resulted in many beneficiaries choosing different nursing homes than they would have when using the standard rating system. Given the substantial variation in medical needs of patients and their preferences for what constitutes high-quality hospital care, beneficiaries may benefit from personalized star-rating systems.

Past research has demonstrated that many Medicare hospitals that receive high- or low-quality ratings are not significantly different than average hospitals.^29^ Given how strong an influence that quality ratings appear to have on patients' choice of provider, it is vital that the differences in quality ratings reflect meaningful differences in outcomes. If not, there is the potential that publicly reported quality metrics could result in beneficiaries making suboptimal decisions, such as a choosing to travel to a more distant hospital despite it providing similar quality to a hospital that is closer to home.

### Limitations

Several limitations merit consideration. First, we included 4 attributes related to hospital quality that were derived from Medicare's Hospital Value-based Purchasing Program. There are many ways of conceptualizing hospital quality, which can include a different number or complement of quality domains, and our conceptualization may have failed to include some aspects of quality that matter to some patients. That said, we used quality domains derived from Medicare's Hospital Value-based Purchasing Program given the potential to inform policy decisions. Second, we only fielded the survey in English. Thus, while our weighted sample was broadly representative of the Medicare population with respect to race and ethnicity, our results do not necessarily represent the preferences of non-English speakers. Third, we chose not to specify which condition that respondents were seeking care for. Given that the value assigned to different aspects of quality may vary based on the severity of the underlying condition, our estimates likely underestimate WTP/WTT for more severe health conditions and overestimate it for less severe conditions. Fourth, our WTP/WTT estimates represent mean estimates across a national sample, and we would expect these estimates to vary given preference heterogeneity, both at the individual level and among subgroups of the population. Last, it is unclear how well our results reflect real-world decision making, and the extent to which publicly reported quality ratings impact decisions relative to other aspects of the decision-making process, such as the recommendation of a friend, family member, or doctor. As described above, while there is strong evidence that higher-quality hospitals attract a greater market share, it is unclear what is driving this. Research is needed to better understand the decision-making process and the extent to which the stated preferences we report are reflected in real-world decisions.

## Conclusion

In this study we have quantified how much Medicare beneficiaries might be willing to pay or how far they might be willing to travel to access hospitals with higher quality ratings. Our results suggest that, on average, clinical outcomes is the most important quality domain. However, the value of a 1-star improvement depends not only on the quality domain but also on the baseline level of performance of the hospital. Generally, it is more valuable for low-performing hospitals to achieve average performance than for average hospitals to achieve excellence (ie, there are diminishing marginal returns). These findings may help physicians and policymakers understand the diverse value of different aspects of quality to Medicare beneficiaries, which, in turn, can support quality-reporting and -improvement activities.

## Supplementary Material

qxad085_Supplementary_Data
