# Supplementary material for: What is a star worth to Medicare beneficiaries? A discrete choice experiment of hospital quality ratings
Source: Health Aff Sch. 2023 Dec 12;2(1):qxad085. doi: 10.1093/haschl/qxad085 (PMC10986207; doi:10.1093/haschl/qxad085)
Supplement: qxad085_Supplementary_Data [file qxad085_Supplementary_Data.zip › Trenaman_Supplemental_Materials.pdf]

Supplemental Material

- Table S1: Experimental design
- Table S2. Characteristics of the unweighted and weighted samples and the Medicare population
- Table S3. Medicare beneficiaries' willingness to pay (WTP) for hospital quality ratings, model 1 (continuous)
- Table S4. Medicare beneficiaries' willingness to travel (WTT) for hospital quality ratings, model 1 (continuous)
- Figure S1. Medicare beneficiaries' willingness to pay (WTP) and willingness to travel (WTP) for hospital quality ratings, model 2 (categorical)
- Table S5. Medicare beneficiaries' willingness to pay (WTP) for hospital quality ratings, model 2 (categorical)
- Table S6. Medicare beneficiaries' willingness to travel (WTT) for hospital quality ratings, model 2 (categorical)

Table S1. Experimental Design

Clinical Outcomes	Patient Experience	Safety	Efficiency	Distance	Out of Pocket Cost	Choice Set	Alt	Block
5	4	3	3	3	1	1	1	1
2	1	5	2	1	3	1	2	1
1	2	3	4	2	1	2	1	3
5	5	1	3	1	3	2	2	3
3	1	5	4	2	3	3	1	2
1	3	1	2	1	1	3	2	2
1	1	3	2	2	3	4	1	5
3	3	4	4	3	2	4	2	5
1	5	5	4	3	2	5	1	3
3	1	1	3	1	3	5	2	3
5	1	2	5	2	2	6	1	3
4	4	3	2	1	3	6	2	3
4	3	5	4	3	1	7	1	1
2	4	4	5	1	2	7	2	1
4	2	2	2	3	2	8	1	2
3	5	5	5	1	1	8	2	2
4	1	3	3	1	1	9	1	3
2	4	2	1	3	3	9	2	3
5	2	5	2	1	2	10	1	2
4	5	3	5	2	3	10	2	2
3	5	1	1	1	3	11	1	2
2	4	5	3	3	1	11	2	2
1	1	4	2	3	3	12	1	4
4	3	5	1	1	2	12	2	4
4	2	1	2	3	2	13	1	5
1	1	2	4	1	3	13	2	5
5	5	3	3	2	1	14	1	4
4	1	1	1	3	2	14	2	4
2	5	1	3	3	1	15	1	5
1	4	3	5	1	2	15	2	5

3	4	5	3	3	1	16	1	3
5	2	4	4	1	2	16	2	3
5	4	5	5	1	2	17	1	5
3	3	4	1	2	1	17	2	5
3	2	3	2	1	1	18	1	1
4	1	4	3	2	2	18	2	1
1	2	2	3	1	2	19	1	3
5	4	4	4	2	3	19	2	3
3	2	1	4	2	2	20	1	1
5	1	5	2	3	3	20	2	1
4	3	2	5	1	1	21	1	4
2	2	5	3	3	3	21	2	4
3	2	2	5	2	3	22	1	5
2	5	4	1	1	2	22	2	5
4	2	1	4	2	3	23	1	3
3	3	3	1	3	2	23	2	3
2	2	3	1	1	3	24	1	2
4	1	4	2	2	2	24	2	2
1	3	4	3	2	1	25	1	1
4	5	3	4	3	2	25	2	1
1	5	2	4	3	1	26	1	1
3	1	3	5	2	2	26	2	1
2	3	2	5	2	3	27	1	4
3	1	5	2	1	1	27	2	4
5	3	5	4	2	3	28	1	4
2	4	2	3	1	2	28	2	4
1	5	4	1	2	2	29	1	5
2	3	2	5	3	3	29	2	5
5	4	3	3	3	2	30	1	1
2	2	5	5	1	1	30	2	1
5	3	1	4	1	2	31	1	5
4	2	4	1	3	3	31	2	5
5	2	4	2	2	1	32	1	4

1	1	1	4	3	2	32	2	4
1	5	1	5	3	3	33	1	3
2	1	2	1	2	1	33	2	3
1	2	5	3	2	2	34	1	5
5	1	3	1	3	1	34	2	5
5	2	1	1	3	1	35	1	1
2	5	4	2	1	3	35	2	1
4	5	2	3	1	1	36	1	2
1	3	1	2	3	3	36	2	2
3	4	4	5	3	1	37	1	2
4	3	3	3	1	3	37	2	2
3	3	2	2	2	3	38	1	4
1	1	1	5	3	1	38	2	4
1	4	1	1	2	1	39	1	2
3	2	4	5	3	2	39	2	2
3	4	2	4	1	3	40	1	1
2	3	3	5	2	2	40	2	1
2	4	1	4	2	2	41	1	3
4	5	2	5	3	1	41	2	3
4	4	5	1	2	3	42	1	5
2	1	3	4	3	1	42	2	5
4	4	4	4	1	1	43	1	4
5	5	2	1	2	2	43	2	4
5	2	4	1	1	3	44	1	4
3	5	2	2	2	2	44	2	4
2	5	1	2	2	1	45	1	2
1	3	4	3	3	3	45	2	2

Table S2. Characteristics of the unweighted and weighted samples and the Medicare population

Characteristic	Unweighted Sample	Weighted Sample	Medicare Population*
	%	%	%
**Age group**			
18-64	15%	13%	12%
65-74	55%	50%	50%
75+	31%	37%	37%
**Gender**			
Female	51%	55%	55%
Male	49%	45%	45%
**Ethnicity**			
Black, Non-Hispanic	20%	11%	10%
Hispanic	20%	9%	9%
Other, Non-Hispanic	6%	5%	5%
White, Non-Hispanic	52%	75%	74%
2+ Races, Non-Hispanic	1%	1%	1%
**Highest educational attainment**			
No high school diploma or GED	7%	11%	11%
High school graduate or equivalent	27%	32%	31%
Some college or Associate's degree	32%	26%	24%
Bachelor's degree or higher	34%	31%	28%
**Household income, $**			
Under $25,000	16%	24%	24%
$25,000 to $49,999	24%	25%	25%
$50,000 to $74,999	19%	17%	17%
$75,000 to $99,999	13%	11%	11%
$100,000 to $149,999	14%	12%	12%
$150,000 or more	13%	12%	12%
**Metro**			
Metro	88%	83%	83%
Non-Metro	12%	17%	17%
**Region**			
Midwest	18%	21%	21%
Northeast	16%	18%	18%
South	43%	39%	39%
West	23%	22%	22%

*From the 2021 Current Population Survey data for all Medicare enrollees aged 18 and older

Table S3. Medicare beneficiaries' willingness to pay (WTP) for hospital quality ratings, Model 1 (continuous*)

	Mean			SD		
	**β**	**2.5%**	**97.5%**	**β**	**2.5%**	**97.5%**
**Clinical Outcomes**	$1698	$1483	$1912	$1051	$871	$1231
**Patient Experience**	$691	$587	$794	$448	$181	$715
**Safety**	$615	$497	$733	$609	$488	$731
**Efficiency**	$218	$133	$302	$295	$68	$523
**Distance (per 100 miles)**	-$825	-$997	-$652	$1309	$1004	$1613

Observations: 18,450
Log-likelihood: -4369.48
Akaike information criterion: 8762.97
Bayesian information criterion: 8856.84

* *Estimates indicate how much respondents would be willing to pay, on average, for a hospital with a one-star higher quality rating.*

Table S4. Medicare beneficiaries' willingness to travel (WTT) for hospital quality ratings, model 1 (continuous*)

	Mean			SD		
	**β**	**2.5%**	**97.5%**	**β**	**2.5%**	**97.5%**
**Clinical Outcomes**	185	155	215	128	105	150
**Patient Experience**	77	63	91	47	30	65
**Safety**	62	49	76	45	24	66
**Efficiency**	20	12	28	20	-3	44
**Cost (per $1,000)**	-109	-127	-90	97	80	115

Observations: 18,450
Log-likelihood: -4246.65
Akaike information criterion: 8517.30
Bayesian information criterion: 8611.17

* *Estimates indicate how far respondents would be willing to travel, on average, for a hospital with a one-star higher quality rating.*

Figure S1. Medicare beneficiaries' willingness to pay (WTP) and willingness to travel (WTP) for hospital quality ratings, model 2 (categorical)

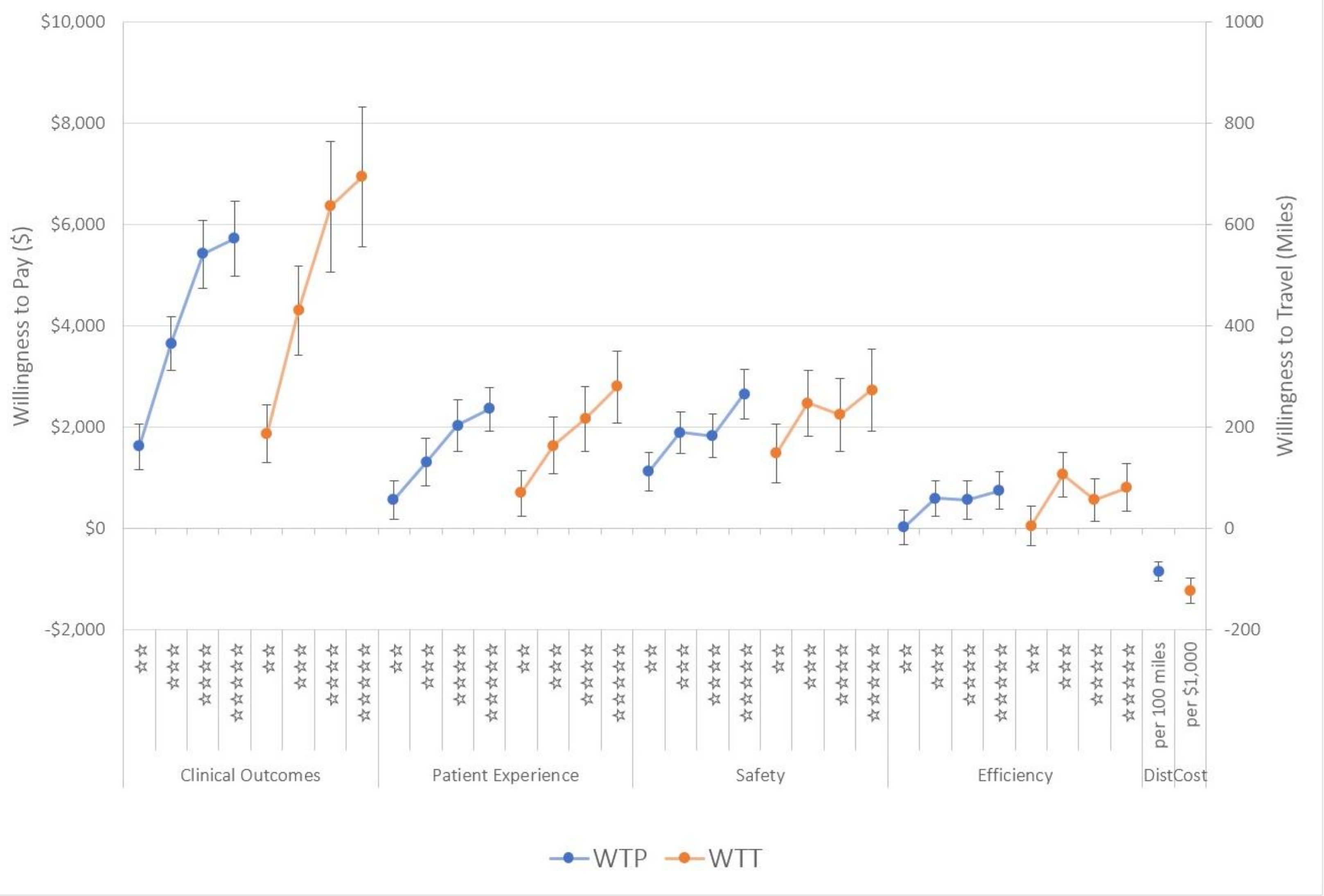

Table S5. Medicare beneficiaries' willingness to pay (WTP) for hospital quality ratings, model 2 (categorical*)

	Mean			SD		
	**β**	**2.5%**	**97.5%**	**β**	**2.5%**	**97.5%**
**Clinical Outcomes**						
One-star	Reference					
Two-star	$1618	$1164	$2072	$1516	$901	$2131
Three-star	$3654	$3120	$4187	$80	-$128	$288
Four-star	$5419	$4750	$6088	$20	-$211	$250
Five-star	$5720	$4984	$6457	$439	-$875	$1753
**Patient Experience**						
One-star	Reference					
Two-star	$563	$188	$938	$537	-$17	$1092
Three-star	$1312	$844	$1780	$1259	$342	$2176
Four-star	$2041	$1528	$2553	$719	-$1212	$2650
Five-star	$2361	$1934	$2788	$217	-$254	$687
**Safety**						
One-star	Reference					
Two-star	$1131	$751	$1510	$642	$61	$1223
Three-star	$1898	$1481	$2314	$41	-$301	$383
Four-star	$1833	$1395	$2271	$673	-$279	$1626
Five-star	$2655	$2157	$3153	$1095	$218	$1672
**Efficiency**						
One-star	Reference					
Two-star	$23	-$319	$365	$77	-$278	$433
Three-star	$600	$254	$947	$233	-$49	$516
Four-star	$561	$178	$945	$78	-$457	$613
Five-star	$747	$374	$1119	$22	-$456	$499
**Distance (per 100 miles)**	-$849	-$1038	-$660	$1433	$1151	$1715

Observations: 18,450
Log-likelihood: -4387.57
Akaike information criterion: 8847.14
Bayesian information criterion: 9128.76

** Estimates indicate how much respondents would be willing to pay, on average, for a hospital with that star rating, relative to a one-star hospital.*

Table S6. Medicare beneficiaries' willingness to travel (WTT) for hospital quality ratings, model 2 (categorical*)

	Mean			SD		
	**β**	**2.5%**	**97.5%**	**β**	**2.5%**	**97.5%**
**Clinical Outcomes**						
One-star	Reference					
Two-star	187	129	244	150	55	244
Three-star	431	343	519	9	-14	32
Four-star	636	507	765	27	-60	115
Five-star	695	556	833	139	60	218
**Patient Experience**						
One-star	Reference					
Two-star	70	25	115	10	-79	99
Three-star	164	107	220	53	-12	117
Four-star	217	154	281	108	15	200
Five-star	280	210	351	76	-11	164
**Safety**						
One-star	Reference					
Two-star	149	91	207	89	3	175
Three-star	248	182	313	13	-25	51
Four-star	225	154	297	123	60	185
Five-star	274	193	355	145	72	218
**Efficiency**						
One-star	Reference					
Two-star	5	-35	44	27	-17	70
Three-star	107	63	151	64	2	125
Four-star	57	15	99	24	-19	66
Five-star	81	34	128	18	-28	64
**Cost (per $1000)**	-123	-149	-98	115	88	142

Observations: 18,450
Log-likelihood: -4303.72
Akaike information criterion: 8679.44
Bayesian information criterion: 8961.06

* *Estimates indicate how far respondents would be willing-to-travel, on average, for a hospital with that star rating, relative to a one-star hospital.*
